# Speaking in Alzheimer’s Disease, is That an Early Sign? Importance of Changes in Language Abilities in Alzheimer’s Disease

**DOI:** 10.3389/fnagi.2015.00195

**Published:** 2015-10-20

**Authors:** Greta Szatloczki, Ildiko Hoffmann, Veronika Vincze, Janos Kalman, Magdolna Pakaski

**Affiliations:** ^1^Research Institute for Linguistics, Hungarian Academy of Sciences, Szeged, Hungary; ^2^Research Institute for Linguistics, Hungarian Academy of Sciences, Budapest, Hungary; ^3^Department of Linguistics, University of Szeged, Szeged, Hungary; ^4^MTA-SZTE Research Group on Artificial Intelligence, University of Szeged, Szeged, Hungary

**Keywords:** screening, mild cognitive impairment, Alzheimer’s disease, language domain, systematic review

## Abstract

It is known that Alzheimer’s disease (AD) influences the temporal characteristics of spontaneous speech. These phonetical changes are present even in mild AD. Based on this, the question arises whether an examination based on language analysis could help the early diagnosis of AD and if so, which language and speech characteristics can identify AD in its early stage. The purpose of this article is to summarize the relation between prodromal and manifest AD and language functions and language domains. Based on our research, we are inclined to claim that AD can be more sensitively detected with the help of a linguistic analysis than with other cognitive examinations. The temporal characteristics of spontaneous speech, such as speech tempo, number of pauses in speech, and their length are sensitive detectors of the early stage of the disease, which enables an early simple linguistic screening for AD. However, knowledge about the unique features of the language problems associated with different dementia variants still has to be improved and refined.

## Introduction

Despite great efforts concentrated on disease modifying therapies of Alzheimer’s disease (AD), halting the degenerative process has not been possible. For this reason, early diagnosis of AD became crucial in the management of the disease. Current pharmacological agents available for AD are more effective in the mild cases, even in the cases of mild cognitive impairments (MCI). It is well-documented that manifest AD patients show markers of language deficit long before their diagnosis is confirmed (Mesulam et al., 2008) and this tendency is especially useful for detecting mild cognitive decline, the prodromal stage of AD (Garrard et al., 2005).

Diagnostic procedures of language functions play a major role in the detection process of the cognitive deficits with different stages. Questions nevertheless remain whether the characterization of the linguistic profiles of MCI/AD cases is useful or not in the detection procedure. The purpose of this review is to summarize the main language deficits in relation to prodromal and manifest AD, focusing on the changes of different language domains (semantic, pragmatic, syntactic, and phonologic ones) during the course of the disease. Additionally, the relationship between language and other cognitive functions in AD will be discussed.

## Alzheimer’s Disease and Language

Cognitive deficits involve executive function, reasoning, visuoconstructive, and language abilities. Language deficits typically become noticeable from the early stage of the disease (Morris, 1996). Naming disorders, impaired auditory and written comprehension, fluent but empty speech, and semantic paraphasia are typical language deficits in AD, however, repetition abilities and articulation remain relatively intact (Appell et al., 1982; Bayles et al., 1992; Croot et al., 2000). The different stages of the disease exhibit specific patterns of linguistic difficulties in a given domain. The following five domains of language are known: phonetics and phonology, morphology, lexicon and semantics, syntax, and pragmatics. These language domains are affected in different ways in AD (Bayles and Boone, 1982).

In the Table 1, we are going to summarize the language function measurements of MCI and different stages in AD. As the disease progresses (from MCI to severe AD), a continuous decline in language can be observed in AD patients (Kempler, 2004).

**Table 1:** **Alteration in MCI and ad concerning phonetics, phonology, lexicon, semantics, and pragmatics**.

Examination methods	Examination results	Sensitivity measures	Reference
**Phonetics and phonology**			
Temporal analysis of spontaneous speech	Mild AD and CTRL differ in speech tempo and hesitation ratio	No data	Hoffmann et al. (2010)
Temporal analysis of speech, oral reading task	Distinguishes moderate AD and CTRL. Best two parameters: speech tempo and articulation tempo	80%	Martínez-Sánchez et al. (2013)
Spoken task; speech-based detection	Might be a good method for detecting early AD	CTRL and MCI: 80%	Satt et al. (2014)
		MCI and AD: 87%	
Automatic spontaneous speech analysis	Distinguishes between AD and CTRL	No data	López-de-Ipiña et al. (2013)
**Lexicon, semantics and pragmatics**			
Semantic association test	AD performs significantly worse than CTRL	No data	Visch-Brink et al. (2004)
Semantic verbal fluency and phonological verbal fluency	Good tool for diagnosis of early AD	No data	Laws et al. (2010)
Picture naming, semantic probes, lexical decision and priming, Stroop-picture naming	AD group was impaired in semantic tasks	No data	Duong et al. (2006)
Verbal task	AD group produces shorter texts, less relevant information and multiple error types than CTRL	No data	Taler and Phillips (2008)

*AD, Alzheimer’s disease; MCI, mild cognitive impairment; CTRL, healthy controls*.

## The Relationship Between Language and Cognitive Functions in Alzheimer’s Disease

In AD, language and memory functions are closely related since linguistic functioning requires memory functions. Difficulties in productive speech, speech comprehension, and memory functions overlap. Senile changes in language comprehension and expression entail the decline of global speech performance, and a lapse in evocative memory puts constraints on the active vocabulary (Kempler, 2004).

In a summative work, the relationship between simple language measures and cognitive impairment in AD was estimated by the mini-mental state examination (MMSE) and the clinical dementia rating scale (CDR), respectively. Language measures included articulation, fluency (word-finding ability, hypofluency, hyperfluency), semantic fluency, repetition, and confrontational naming. A significant relationship was found between CDR and MMSE scores and all language measures apart from hyperfluency. Impairment in language fluency, animal naming, and confrontational naming are common, especially in the case of impaired cognitive and global performance (Weiner et al., 2008).

It has also been shown that patients with AD show difficulties in performing tasks that tap semantic knowledge, such as naming, verbal fluency, or object recognition. These symptoms occur early and they increase during the course of the illness, suggesting early and progressive impairment of the semantic memory of these patients (Nebes et al., 1989). Briefly, semantic memory can be defined as the capacity to acquire and retain general knowledge about the world, containing basic facts and meanings, as well as words and their meanings. Several approaches have been put forward in order to test semantic memory, such as priming tests, category fluency, and object or picture naming (Hodges, 1994).

Another stream of research aims at the examination of lexical semantic memory (Balthazar et al., 2007). According to these results, the three groups (control, amnestic MCI, mild AD) showed a continuum of decreasing cognitive ability in all cognitive tests. In semantic memory tests, the performance of amnestic MCI patients was similar to that of controls, but showed worse results on verbal fluency task, which involves semantic knowledge, as well as language use, executive function, and short-term memory. Thus, verbal fluency might have been influenced by short-term memory. As the disease progresses, other areas including the temporal cortex are involved, which can explain the difficulties with semantic knowledge in mild AD. It has been shown that amnestic MCI impairs episodic memory while the lexical semantic system is spared, which can be affected in the early phase of AD.

In summary, deficits in language and memory functions, especially in semantic memory are commonly found in patients with AD, even in the early phase. Therefore, the need can arise for developing a purely language-based screening test, which can serve as an early diagnostic tool for MCI.

## Neural Bases of Language Deficits in Alzheimer’s Disease

Considering the cognitive impairments in AD, the neural basis of episodic memory has been primarily investigated by the anatomical and functional neuroimaging techniques, such as functional magnetic resonance imaging (fMRI), diffusion tensor imaging (DTI), or positron emission tomography (PET). So far, only a limited number of publications are available, which focus on the detection of organic or functional changes in the central nervous system underlying language impairments. For example, a recent investigation of healthy subjects and individuals with amnestic mild cognitive impairment (aMCI) demonstrated a difference in the neuroanatomical bases of episodic and semantic performance (Hirni et al., 2013). Specifically, region of interest (ROI) analyses showed that episodic memory performance was associated with the bilateral entorhinal cortex/hippocampus (ERC/HP) head, whereas semantic memory performance was associated with left medial perirhinal cortex (mPRC) and bilateral ERC/HP head integrity suggesting that mPRC damage in very early AD may be detectable with common clinical tests of semantic memory if episodic memory performance is controlled (Hirni et al., 2013).

In another study, a 2-back versus 1-back letter recognition task was performed by MCI and AD patients, using DTI and fMRI. Significant hypoactivation was found in posterior brain areas and relative hyperactivation in anterior brain areas during working memory in AD/MCI subjects compared to controls. In MCI/AD subjects, impairments of structural fiber tract integrity co-occur with breakdown of posterior and relatively preserved anterior cortical activation during working memory performance (Teipel et al., 2014).

Posterior corpus callosum connects superior parietal, posterior temporal, and occipital cortical areas (De Lacoste et al., 1985), which include key nodes of working memory activation. The superior longitudinal fasciculus forms a large arc superior and lateral to the putamen connecting all four cerebral lobes, which has a main role in language processing in the human brain (Bernal and Altman, 2010; Axer et al., 2013). This area is known to be impaired in MCI and mild AD (Liu et al., 2011; Zhang et al., 2013) and is a possible reason for functional uncoupling of prefrontal and posterior brain areas during verbal working memory performance (Teipel et al., 2014).

## Language Functions during the Course of Alzheimer’s Disease

The impairment of the language functions in the course of AD may be characteristic not only for the given stage of the disease but also for its prodroma, MCI. During the total course of the disease, language seems to be impaired disproportionally, meaning that the semantic and pragmatic language systems are more impaired than syntax (Bayles and Boone, 1982). Impairments in the lexical, semantic, and pragmatic language functions are typically present in mild AD since they depend on cognition to a greater extent (Taler and Phillips, 2008; Tsantali et al., 2013). Articulatory and syntactic domains of language production remain intact until late stages of the disease (Croot et al., 2000).

In the following sections, relevant studies will be discussed and summarized in order to investigate language functioning during the course of AD, considering the most extensively researched language domains (Table 2).

**Table 2:** **Language functions in mild cognitive impairment and in the different stages of Alzheimer’s disease**.

Language characteristic changes		MCI	Mild AD	Moderate AD	Severe AD	Reference
**Phonetics-phonology**						
Temporal changes in spontaneous speech (increasing hesitation number and time)		+	+	++	+++	Forbes and Venneri (2005); Hoffmann et al. (2010); Roark et al. (2011); Meilán et al. (2012); Satt et al. (2014); Jarrold et al. (2014); Laske et al. (2015)
Phonemic paraphasia		+	+	++	+++	Croot et al. (2000); Forbes et al. (2002); Hoffmann et al. (2010); Wutzler et al. (2013); Roark et al. (2011); Satt et al. (2014); Jarrold et al. (2014)
**Lexical-semantics**						
Word-finding and word retrieval difficulties		+	+	++	+++	Smith et al. (1989); Bayles (1993); Light (1993); Kempler and Zelinski (1994); Kempler et al. (2001); Garrard et al. (2005); Taler and Phillips (2008); Dos Santos et al. (2011); Cardoso et al. (2014); Fraser et al. (2014); Laske et al. (2015); Garrard et al. (2014)
Verbal fluency difficulties	Phonemic (letter)	+	+	++	+++	Barth et al. (2005); Juncos-Rabadán et al. (2010); Hoffmann et al. (2010); Dos Santos et al. (2011); Roark et al. (2011); Satt et al. (2014); Jarrold et al. (2014)
	Semantic	+	+	++	+++	
Semantic paraphasia		?	+	++	+++	Juncos-Rabadán et al. (2010); Hoffmann et al. (2010); Roark et al. (2011); Satt et al. (2014); Jarrold et al. (2014)
**SYNTAX**						
Reduced syntactic complexity		−	−	+	+++	Caramelli et al. (1998); Small et al. (1997); Kempler (1995); Bickel et al. (2000); Ullman (2001); Juncos-Rabadán et al. (2010)
Agrammatisms		−	−	−	+++	Small et al. (1997); Kempler (1995); Ullman (2001)
**DISCOURSE-PRAGMATICS**						
Reduction in productive and receptive discourse-level processing		−/+	+	++	+++	Hodges et al. (1992); Ripich (1994); Taler and Phillips (2008); Weiner et al. (2008); Hoffmann et al. (2010); Juncos-Rabadán et al. (2010); Rapp and Wild (2011); Tsantali et al. (2013); Cardoso et al. (2014)

*AD, Alzheimer’s disease; MCI, mild cognitive impairment*.

*The scale of MMSE scores is as follows: MCI: 28–26 points (Roalf et al., 2013), mild AD: 25–20 points, moderate AD: 19–10 points, and severe AD: 9–0 points (Vertesi et al., 2001)*.

*+, degree of involvement; −, intact; ?, no data*.

### Phonetics and Phonology in Alzheimer’s Disease

Temporal parameters of speech can be investigated in the language domains phonetics and phonology, more precisely, in spontaneous speech (Hoffmann et al., 2010; López-de-Ipiña et al., 2013), in a reading aloud task (Martínez-Sánchez et al., 2013), and in spoken tasks (Satt et al., 2014).

In the MCI phase, the most characteristic linguistic changes are longer hesitations and a lower speech rate in spontaneous speech (Hoffmann et al., 2010; Roark et al., 2011; Jarrold et al., 2014; Satt et al., 2014). The manually extracted acoustic features of spontaneous speech and an automatizing biomarker extraction process using automatic speech recognition (ASR) have been recently compared in MCI patients and control subjects (Tóth et al., 2015). The classification results provided by ASR-based feature extraction were just slightly worse than those of the manual method (Tóth et al., 2015).

The temporal parameters of spontaneous speech have also been investigated in mild AD and control subjects (Hoffmann et al., 2010). This study aimed to identify a speech parameter that might distinguish mild AD patients from normal individuals. The following aspects of spontaneous speech were included in the analysis: articulation rate, speech tempo, hesitation ratio, and grammatical error ratio. Results showed that articulation rate in mild and severe AD patients was significantly different from normal controls; furthermore, a difference among mild, moderate, and severe AD patients was also reported. Significant differences in speech tempo and hesitation ratio were found between all experimental groups, apart from moderate and severe AD patients, who performed similarly on both tasks. Grammatical error analysis showed significant difference between moderate and severe AD groups; however, this was not found when comparing normal subjects and mild AD groups (Hoffmann et al., 2010).

In another study, an automatic spontaneous speech analysis was also carried out to identify mild AD. It was suggested that shorter recording times reflect that for AD patients, speech requires more efforts than for healthy individuals: patients speak more slowly with longer pauses, as well as they spend more time to find the correct word, which in turn leads to speech disfluency or break messages (López-de-Ipiña et al., 2013).

A similar research studied the temporal organization of speech in AD patients and matched healthy controls with an oral reading task. The following indices were analyzed: total duration of the reading task, number of pauses, pause proportion, phonation time, phonation – time ratio, speech rate, and articulation rate. The AD group showed impairment in all of these variables. Reduced speech and articulation rates, low effectiveness of phonation time, as well as increased number and proportion of pauses characterized their reading. The two temporal parameters with the greatest discriminatory capacity were speech rate and articulation rate. In sum, signal processing algorithms applied to reading fluency recordings were capable of differentiating between AD patients and controls with an accuracy of 80% based on speech rate. Thus, analyzing temporal parameters for reading fluency, especially speech and articulation rates, allowed to distinguish between asymptomatic subjects and patients in mild AD (Martínez-Sánchez et al., 2013).

Although examining the temporal parameters of spontaneous speech, it is not clear which variables are capable of separating the mild AD group from the control group. Some researchers divided the mild AD group from the control group based on the articulation rate, speech tempo, and hesitation ratio variables (Hoffmann et al., 2010), whereas others suggested that speech rate and articulation rate are the best discriminating variables (Martínez-Sánchez et al., 2013). Furthermore, some researchers emphasize the importance of break analysis as well (López-de-Ipiña et al., 2013). However, there is an agreement that the temporal analysis of spontaneous speech is proven to be an effective method for spotting mild AD.

In moderate or severe AD, there are more and more serious temporal changes in spontaneous speech: hesitation number and time increase, compared to mild AD, and the mental lexicon is even more difficult to access (Hoffmann et al., 2010).

### Lexical, Semantic, and Pragmatic Domains of Language in Alzheimer’s Disease

Mild cognitive impairment patients usually have trouble with finding the right word (Fraser et al., 2014; Garrard et al., 2014). As regards semantics and syntax, both seem to be impaired since fluency tasks and naming tasks show deficits; moreover, comprehension of sentences and texts and production of narrative speech are also impaired, concerning the semantic content and syntactic structures of speech (Juncos-Rabadán et al., 2010).

Alzheimer’s disease patients lack the distinctive semantic attributes of concepts: there is strong evidence that dysfunction in linguistic tasks is caused by the general cognitive impairment in AD (Feinberg and Farah, 1997). The most common and obvious language errors made by AD patients are semantic errors (Croot et al., 2000), namely that they use superordinate category names instead of the target name (Saito and Takeda, 2001) or circumlocutory speech with progressively impaired naming (Emery, 2000).

The semantic association test (SAT) is a tool for detecting disorders in verbal and visual semantic processing (Visch-Brink and Denes, 1993). In general, AD patients had significantly lower scores on SAT than controls. However, their data expose an incoherent relation between naming and semantic processing in AD. In contrast to semantic processing, the performance of AD patients on naming fell within the normal range, implying that naming is independent of semantic processing in AD (Visch-Brink et al., 2004).

Alzheimer’s disease patients typically have difficulties in tasks of confrontational naming and verbal fluency (Appell et al., 1982; Bayles et al., 1987). Semantic verbal fluency and phonological verbal fluency tests are widely used in diagnosis of AD and they are reliable indicators of language deterioration in the early detection of AD (Laws et al., 2010). Difficulties in word finding are one of the earliest manifestations of language breakdown in AD. This pattern of impairment has been implicated as the loss of semantic knowledge in AD (Hodges et al., 1992). Results from language tests and priming experiments clearly suggest altered intentional and automatic semantic processes in AD. However, the order in which these processes are impaired during the course of the disease is unclear (Duong et al., 2006).

Lexico-semantic impairments in AD have been attributed to abnormalities in intentional and automatic access to semantic memory. In a study, MCI, pre-AD, and normal elderly people were tested with intentional access tasks (picture naming and semantic probes), automatic access tasks (lexical decision and priming), and executive function tasks (Stroop and Stroop-picture naming). Results indicated that the MCI group was only impaired in tasks of intentional access relative to the AD group, which showed impairment in all tasks. Since most MCI subjects eventually develop AD, the results suggest that the intentional access to semantic memory is impaired earlier compared to the automatic access. The AD individuals performed significantly different from normal controls in all four semantic tasks (Duong et al., 2006). AD subjects demonstrated slowing in lexical decision as well as increase in semantic priming, termed hyperpriming (Giffard et al., 2001, 2002), which speaks for abnormal automatic semantic processing. Abnormal performance has also been found in picture naming and semantic probe questions which require effortful semantic processing and search. The results confirmed the observation that subtle cognitive impairments, such as language impairment, may co-occur with the readily observed memory impairments (Petersen et al., 1999, 2001; Ritchie et al., 2001).

Alterations in productive and receptive discourse-level processing have also been reported in MCI and mild AD. AD individuals generally produce shorter texts than the normal controls with less relevant information and multiple error types (incoherent/indefinite phrases, semantic and graphemic paraphasia, and inability to abstract) and describe all pictorial themes (Taler and Phillips, 2008).

To sum up, we can say that the performance of AD patients is different compared to the control group in most of the semantic tasks. Changes in semantic processing (Petersen et al., 1999, 2001; Ritchie et al., 2001; Duong et al., 2006) trigger semantic errors in AD patients (Croot et al., 2000). Furthermore, impaired naming (Emery, 2000) and picture naming (Petersen et al., 1999, 2001; Ritchie et al., 2001), word finding difficulties, and abnormal verbal fluency are also present in this group (Appell et al., 1982; Bayles et al., 1987). Slow lexical decision could be one of the reasons behind all of these (Giffard et al., 2001, 2002). However, it should be noted that although lexico-semantic changes in AD have been intensively studied, research on pragmatics has rarely been carried out among AD patients, thus it constitutes a potential field for future investigations.

## Conclusion

On the basis of the existing research findings, we can state that the language deficit in AD is present in the early stage of the disease; therefore, the objective measures of the different language domains are very important in the recognition of these patients. However, up to now, very few linguistic methods have been published, which are suitable for the early diagnosis of AD.

The disproportional impairments of language functions in the course of the disease have been proven by almost cohort studies. Large scale prospective longitudinal studies would be more beneficial; however, they have been also missing. Additionally, more extensive use of functional neuroimaging techniques based on linguistic tasks in MCI or mild AD could lead to a more informed picture of the neural bases of language functions in the different stages of the disease.

In the future, additional work needs to be done to validate new methods across different settings (such as population-based, primary care, and memory clinics), age, and ethnic groups. Since the earliest measurable language domain is the temporal parameter of speech, the computerized analysis of spontaneous speech developed recently may be a promising approach in the early detection of AD. The combined use of the measurement of linguistic parameters and telemedicine technologies might permit the screening of MCI or mild AD by an interactive test using a software package or mobile application. Having an accurate method to assess for dementia and predict risk in routine clinical care will aid decision-making and can ultimately lead to disease prevention.

## Conflict of Interest Statement

The authors declare that the research was conducted in the absence of any commercial or financial relationships that could be construed as a potential conflict of interest.

## References

[B1] Appell J. Kertesz A. Fishman M. (1982). A study of language functioning in Alzheimer patients. Brain Lang. 17, 73–91. 10.1016/0093-934X(82)90006-2 7139272

[B2] Axer H. Klingner C. M. Prescher A. (2013). Fiber anatomy of dorsal and ventral language streams. Brain Lang. 127, 192–204. 10.1016/j.bandl.2012.04.015 22632814

[B3] Balthazar M. L. F. Martinelli J. E. Cendes F. Damasceno B. P. (2007). Lexical semantic memory in amnestic mild cognitive impairment and mild Alzheimer’s disease. Arquivos de Neuropsiquiatria 65, 619–622. 10.1590/S0004-282X2007000400014 17876402

[B4] Barth S. Schönknecht P. Pantel J. Schröder J. (2005). Mild cognitive impairment and Alzheimer’s disease: an investigation of the CERAD-NP test battery. Fortschr. Neurol. Psychiatr. 73, 568–576. 10.1055/s-2004-830249 16217697

[B5] Bayles K. A. (1993). “Pathology of language behaviour in dementia,” in Linguistic Disorders and Pathologies, eds Blamken G. Dittmann J. Grimm H. Marshall J. C. Wallesch C.-W. (Berlin/New York, NY: de Gruyter), 388–409.

[B6] Bayles K. A. Boone D. R. (1982). The potential of language tasks for identifying senile dementia. J. Speech Hear. Disord. 47, 210–217. 10.1044/jshd.4702.210 7176601

[B7] Bayles K. A. Kaszniak A. W. Tomoeda C. K. (1987). Communication and Cognition in Normal Aging and Dementia. Boston, MA: College-Hill Press.

[B8] Bayles K. A. Tomoeda C. K. Trosset W. (1992). Relation of linguistic abilities of Alzheimer’s patients to stage of disease. Brain Lang. 42, 454–472. 10.1016/0093-934X(92)90079-T 1377076

[B9] Bernal B. Altman N. (2010). The connectivity of the superior longitudinal fasciculus: a tractography DTI study. Magn. Reson. Imaging 28, 217–225. 10.1016/j.mri.2009.07.008 19695825

[B10] Bickel C. Pantel J. Eysenbach K. Schröder J. (2000). Syntactic comprehension deficits in Alzheimer’s disease. Brain Lang. 71, 432–448. 10.1006/brln.1999.2277 10716871

[B11] Caramelli P. Mansur L. L. Nitrini R. (1998). “Language and communication disorders in dementia of the Alzheimer type,” in Handbook of Neurolinguistics, eds Stemmer B. Whitaker H. A. (San Diego, CA: Academic Press), 463–473.

[B12] Croot K. Hodges J. R. Xuereb J. Patterson K. (2000). Phonological and articulatory impairment in Alzheimer’s disease: a case series. Brain Lang. 75, 277–309. 10.1006/brln.2000.2357 11049669

[B13] Cardoso S. Silva D. Maroco J. de Mendonca A. Guerreiro M. (2014). Non-literal language deficits in mild cognitive impairment. Psychogeriatr. 14, 222–228. 25495083 10.1111/psyg.12101

[B14] De Lacoste M. C. Kirkpatrick J. B. Ross E. D. (1985). Topography of the human corpus callosum. J. Neuropathol. Exp. Neurol. 44, 578–591. 10.1097/00005072-198511000-00004 4056827

[B15] Dos Santos V. Thomann P. A. Wüstenberg T. Seidl U. Essig M. Schröder J. (2011). Morphological cerebral correlates of CERAD test performance in mild cognitive impairment and Alzheimer’s disease. J. Alzheimers Dis. 23, 411–420. 10.3233/JAD-2010-100156 21116054

[B16] Duong A. Whitehead V. Hanratty K. Chertkow H. (2006). The nature of lexico-semantic processing deficits in mild cognitive impairment. Neuropsychologia 44, 1928–1935. 10.1016/j.neuropsychologia.2006.01.034 16616942

[B17] Emery V. O. B. (2000). Language impairment in dementia of the Alzheimer type: a hierarchical decline? Int. J. Psychiatry Med. 30, 145–164. 10.2190/X09P-N7AU-UCHA-VW08 11001278

[B18] Feinberg T. E. Farah M. J. (1997). Behavioural Neurology and Neuropsychology. New York, NY: McGraw-Hill.

[B19] Forbes K. E. Venneri A. (2005). Detecting subtle spontaneous language decline in early Alzheimer’s disease with a picture description task. Neurol. Sci. 26, 243–254. 10.1007/s10072-005-0467-9 16193251

[B20] Forbes K. E. Venneri A. Shanks M. F. (2002). Distinct patterns of spontaneous speech deterioration: a mild predictor of Alzheimer’s disease. Brain Cogn. 48, 356–361. 12030467

[B21] Fraser K. C. Meltzer J. A. Graham N. L. Leonard C. Hirst G. Black S. E. (2014). Automated classification of primary progressive aphasia subtypes from narrative speech transcripts. Cortex 55, 43–60. 10.1016/j.cortex.2012.12.006 23332818

[B22] Garrard P. Maloney L. M. Hodges J. R. Patterson K. (2005). The effects of very early Alzheimer’s disease on the characteristics of writing by a renowned author. Brain 128, 250–260. 10.1093/brain/awh341 15574466

[B23] Garrard P. Rentoumi V. Gesierich B. Miller B. Gorno-Tempini M. L. (2014). Machine learning approaches to diagnosis and laterality effects in semantic dementia discourse. Cortex 55, 122–129. 10.1016/j.cortex.2013.05.008 23876449 PMC4072460

[B24] Giffard B. Desgranges B. Nore-Mary F. Lalevee C. Beaunieux H. de la Sayette V. (2002). The dynamic time course of semantic memory impairment in Alzheimer’s disease: clues from hyperpriming and hypopriming effects. Brain 125, 2044–2057. 10.1093/brain/awf209 12183350

[B25] Giffard B. Desgranges B. Nore-Mary F. Lalevee C. de la Sayette V. Pasquier F. (2001). The nature of semantic memory deficits in Alzheimer’s disease: new insights from hyperpriming effects. Brain 124, 1522–1532. 10.1093/brain/124.8.1522 11459744

[B26] Hirni D. I. Kivisaari S. L. Monsch A. U. Taylor K. I. (2013). Distinct neuroanatomical bases of episodic and semantic memory performance in Alzheimer’s disease. Neuropsychologia 51, 930–937. 10.1016/j.neuropsychologia.2013.01.013 23369803

[B27] Hodges J. R. (1994). Cognitive Assessment for Clinicians. New York, NY: Oxford University Press, 5–19.

[B28] Hodges J. R. Salmon D. P. Butters N. (1992). Semantic memory impairment in Alzheimer’s disease: failure of access or degraded knowledge? Neuropsychologia 30, 301–314. 10.1016/0028-3932(92)90104-T 1603295

[B29] Hoffmann I. Németh D. Dye C. Pákáski M. Irinyi T. Kálmán J. (2010). Temporal features of spontaneous speech in Alzheimer’s disease. Int. J. Speech Lang. Pathol. 12, 29–34. 10.3109/17549500903137256 20380247

[B30] Jarrold W. Peintner B. Wilkins D. Vergryi D. Richey C. Gorno-Tempini M. L. (2014). “Aided diagnosis of dementia type through computer-based analysis of spontaneous speech,” in Proceedings of CLPsych (Baltimore, MD), 27–37.

[B31] Juncos-Rabadán O. Pereiro A. X. Facal Y. D. Rodríguez N. (2010). Una revisión de la investigación sobre lenguaje en el deterioro cognitivo leve. Revista de Logopedia, Foniatría y Audiología 30, 73–83. 10.1016/S0214-4603(10)70119-4

[B32] Kempler D. (1995). “Language changes in dementia of the Alzheimer type,” in Dementia and communication, ed. Lubinski R. (San Diego, CA: Singular), 98–114.

[B33] Kempler D. (2004). Neurocognitive Disorders in Aging. Thousand Oaks, CA: SAGE.

[B34] Kempler D. Zelinski E. M. (1994). “Language in dementia and normal aging,” in Dementia and normal aging, eds Huppert F. A. Brayne C. O’Connor D. W. (Cambridge: Cambridge University Press), 331–365.

[B35] Kempler S. Marquis J. Thompson M. (2001). Longitudinal change in language production: effects of aging and dementia on grammatical complexity and propositional concent. Psychol. Aging 16, 600–614. 10.1037/0882-7974.16.4.600 11766915

[B36] Laske C. Sohrabi H. R. Frost S. M. de Ipína K. L. Garrard P. Buscema M. (2015). Innovative diagnostic tools for early detection of Alzheimer’s disease. Alzheimers Dement. 11, 561–578. 10.1016/j.jalz.2014.06.004 25443858

[B37] Laws K. R. Duncan A. Gale T. M. (2010). ‘Normal’ semantic-phonemic fluency discrepancy in Alzheimer’s disease? A meta-analytic study. Cortex 46, 595–601. 10.1016/j.cortex.2009.04.009 19560132

[B38] Light L. L. (1993). “Language changes in old age,” in Linguistic Disorders and Pathologies, eds Blanken G. Dittmann J. Grimm H. Marshall J. C. Wallesch C.-W. (Berlin/New York, NY: de Gruyter), 900–918.

[B39] Liu Y. Spulber G. Lehtimaki K. K. Könönen M. Hallikainen I. Gröhn H. (2011). Diffusion tensor imaging and tract-based spatial statistics in Alzheimer’s disease and mild cognitive impairment. Neurobiol. Aging 32, 1558–1571. 10.1016/j.neurobiolaging.2009.10.006 19913331

[B40] López-de-Ipiña K. Alonso J. B. Travieso C. M. Solé-Casals J. Egiraun H. Faundez-Zanuy M. (2013). On the selection of non-invasive methods based on speech analysis oriented to automatic Alzheimer disease diagnosis. Sensor 13, 6730–6745. 10.3390/s130506730 23698268 PMC3690078

[B41] Martínez-Sánchez F. Meilán J. J. G. García-Sevilla J. Carro J. Arana M. J. (2013). Oral reading fluency analysis in patients with Alzheimer disease and asymptomatic control subjects. Neurología 28, 325–331. 10.1016/j.nrl.2012.07.012 23046975

[B42] Meilán J. J. G. Martínez-Sanchez F. Carrol J. Sánchez J. A. Pérez E. (2012). Acoustic markers associated with impairment in language processing in Alzheimer’s disease. Span. J. Psychol. 15, 2081–2090. 10.5209/rev_SJOP.2012.v15.n2.38859 22774422

[B43] Mesulam M. Wicklund A. Johnson N. Rogalski E. Léger G. C. Rademaker A. (2008). Alzheimer and frontotemporal pathology in subsets of primary progressive aphasia. Ann. Neurol. 63, 709–719. 10.1002/ana.21388 18412267 PMC2858311

[B44] Morris R. G. (1996). The Cognitive Neuropsychology of Alzheimer-Type Dementia. New York: Oxford University Press.

[B45] Nebes R. D. Brady C. B. Huff F. J. (1989). Automatic and attentional mechanisms of semantic priming in Alzheimer’s disease. J. Clin. Exp. Neuropsychol. 11, 219–230. 10.1080/01688638908400884 2925832

[B46] Petersen R. C. Doody R. Kurz A. Mohs R. C. Morris J. C. Rabins P. V. (2001). Current concepts in mild cognitive impairment. Arch. Neurol. 58, 1985–1992. 10.1001/archneur.58.12.1985 11735772

[B47] Petersen R. C. Smith G. E. Waring S. C. Ivnik R. J. Tangalos E. G. Kokmen E. (1999). Mild cognitive impairment: clinical characterization and outcome. Arch. Neurol. 56, 303–308. 10.1001/archneur.56.3.303 10190820

[B48] Rapp A. M. Wild B. (2011). Nonliteral language in Alzheimer dementia: a review. JINS. 17, 207–218. 21241530 10.1017/S1355617710001682

[B49] Ripich D. N. (1994). Functional communication with AD patient: a caregiver training program. Alzheimer Dis. Assoc. Disord. 8, 95–109. 10.1097/00002093-199404000-00011 7999352

[B50] Ritchie K. Artero S. Touchon J. (2001). Classification criteria for mild cognitive impairment: a population-based validation study. Neurology 56, 37–42. 10.1212/WNL.56.1.37 11148233

[B51] Roalf D. R. Moberg P. J. Xie S. X. Wolk D. A. Moelter S. T. Arnold S. E. (2013). Comparative accuracies of two common screening instruments for classification of Alzheimer’s disease, mild cognitive impairment, and healthy aging. Alzheimer Dement. 9, 529–537. 10.1016/j.jalz.2012.10.001 23260866 PMC4036230

[B52] Roark B. Mitchell M. Hosom J. P. Hollingshead K. Kave J. (2011). Spoken language derived measures for detecting mild cognitive impairment. IEEE Transaction on Audio, Speech Language Processing 19, 2081–2090. 10.1109/TASL.2011.2112351 PMC3244269 22199464

[B53] Saito A. Takeda K. (2001). Semantic cueing effects on word retrieval in aphasic patients with lexical retrieval deficit. Brain Lang. 77, 1–9. 10.1006/brln.2000.2388 11247652

[B54] Satt A. Hoory R. König A. Aalten P. Robert P. H. (2014). “Speech-based automatic and robust detection of very early dementia,” in Interspeech (Singapore).

[B55] Small J. A. Kemper S. Lyons K. (1997). Sentence comprehension in Alzheimer’s disease. Effects of grammatical complexity, speech rate, and repetition. Psychol. Aging 12, 3–11. 10.1037/0882-7974.12.1.3 9100263

[B56] Smith S. R. Murdoch B. E. Chenery H. J. (1989). Semantic abilities in dementia of the Alzheimer type 1: lexical semantics. Brain Lang. 36, 314–324. 10.1016/0093-934X(89)90084-9 2920289

[B57] Taler V. Phillips N. A. (2008). Language performance in Alzheimer’s disease and mild cognitive impairment: a comparative review. J. Clin. Exp. Neuropsychol. 30, 501–556. 10.1080/13803390701550128 18569251

[B58] Teipel S. Ehlers I. Erbe A. Holzmann C. Lau E. Hauenstein K. (2014). Structural connectivity changes underlying altered working memory networks in mild cognitive impairment: a three-way image fusion analysis. J. Neuroimaging 10, 101. 10.1111/jon.12178 25354135

[B59] Tóth L. Gosztolya G. Vincze V. Hoffmann I. Szatlóczki G. Biró E. (2015). “Automatic detection of mild cognitive impairment from spontaneous speech using ASR,” in Interspeech 2015 Tutorials & Main Conference, Germany: Dresden.

[B60] Tsantali E. Economidis D. Tsolaki M. (2013). Could language deficits really differentiate mild cognitive impairment (MCI) from mild Alzheimer’s disease. Arch. Gerontol. Geriatr. 57, 263–270. 10.1016/j.archger.2013.03.011 23628238

[B61] Ullman M. T. (2001). The declarative/procedural model of lexicon and grammar. J. Psycholinguist. Res. 30, 37–69. 10.1023/A:1005204207369 11291183

[B62] Vertesi A. Lever J. A. Molloy D. W. Sanderson B. Tuttle I. Pokoradi L. (2001). Standardized mini-mental state examination. Use and interpretation. Can. Fam. Physician 47, 2018–2023. 11723596 PMC2018449

[B63] Visch-Brink E. G. Denes G. (1993). “A European base-line test for word-picture processing,” in Developments in the Assessment and Rehabilitation of Brain-Damaged Patients, eds Stachowiak F. J. De Bleser R. Deloche G. Kaschell R. Kremin H. North P. (Tübingen: Gunter Narr Verlag).

[B64] Visch-Brink E. G. Hagelstein M. Middelkoop H. A. M. Cammen T. M. J. (2004). Naming and semantic processing in Alzheimer dementia: a coherent picture? Brain Lang. 91, 11–12. 10.1016/j.bandl.2004.06.101

[B65] Weiner M. F. Neubecker K. E. Bret M. E. Hynan L. S. (2008). Language in Alzheimer’s disease. J. Clin. Psychiatry 69, 1223–1227. 10.4088/JCP.v69n0804 18505305 PMC3177322

[B66] Wutzler A. Becker R. Lammler G. Hayerkamp W. Steinhanen-Thiessen E. (2013). The anticipatory proportion as an indicator of language impairment in early-stage cognitive disorder in the elderly. Dement. Gertatr. Cogn. Disord. 36, 300–309. 10.1159/000350808 24022211

[B67] Zhang Y. Schuff N. Camacho M. Chao L. L. Fletcher T. P. Yaffe K. (2013). MRI markers for mild cognitive impairment: comparisons between white matter integrity and gray matter volume measurements. PLoS ONE 8:e66367. 10.1371/journal.pone.0066367 23762488 PMC3675142

